# A Three-Year Retrospective Study on Survival of Ceramic-Veneered Zirconia (Y-TZP) Fixed Dental Prostheses Performed in Private Practices

**DOI:** 10.1155/2017/9618306

**Published:** 2017-06-20

**Authors:** Veronika Norström Saarva, Göran Bjerkstig, Anders Örtorp, Per Svanborg

**Affiliations:** Department of Prosthodontics/Dental Materials Science, Institute of Odontology, Sahlgrenska Academy, University of Gothenburg, Gothenburg, Sweden

## Abstract

**Objectives:**

The aim of this retrospective study was to evaluate the three-year clinical outcome for ceramic-veneered zirconia fixed dental prostheses (FDPs).

**Methods:**

All patients who were treated with ceramic-veneered zirconia FDPs, in three private practices in Sweden, during the period June 2003 to April 2007 were included. Case records from 151 patients, treated with a total of 184 zirconia FDPs (692 units), were analysed for clinical data. All complications noted in the charts were registered and compared to definitions for success and survival and statistical analysis was performed using the Kaplan-Meier method and a Cox regression model.

**Results:**

In total, 32 FDPs in 31 patients experienced some type of complication (17.4% of FDPs, 20.5% of patients). Core fractures occurred in two (1.1%) FDPs. Two (1.1%) FDPs or 0.6% of units showed adhesive veneer fractures. Cohesive veneer fractures occurred in 10 (5.4%) FDPs (1.6% of units). The three-year cumulative success and survival rates (CSR) were 82.3% and 95.2%, respectively.

**Conclusions:**

Ceramic-veneered zirconia is a promising alternative to metal-ceramic FDPs, even in the posterior area. However, the higher survival rate of metal-ceramic FDPs should be noted and both dentists and patients must be aware of the risks of complications.

## 1. Introduction

The introduction and rapid development of computer-aided design and computer-aided manufacturing (CAD/CAM) technology in prosthetic dentistry have been highly evident in the last decade. Combined with a search for new materials with aesthetic, biocompatible, and high-strength properties, this has contributed to the increasingly frequent use of dental ceramics in general and yttria-stabilized tetragonal zirconia polycrystals (Y-TZP, hereafter called* zirconia*) in particular [1, 2].

Zirconia is a high-strength ceramic material with high fracture toughness, chemical inertness, and decent aesthetic properties, which is used mainly as a core material for single and multiunit fixed dental prostheses (FDPs) and abutments [3, 4]. The high fracture resistance of zirconia is due to the ability of the material to transform from one structural phase to another, when exposed to stress. The phase transformation results in a volume increase in the stress zone, which hinders cracks from growing [5].

Compared to metal-ceramic (MC) FDPs, a zirconia restoration is more aesthetical. Further, its high fracture strength, considerably higher than alumina, enables the manufacture of all-ceramic FDPs. Which may be bilayered, using a veneering ceramic, or monolithic, using only zirconia as a full-contour restoration.

Zirconia restorations for dental replacements are made using CAD/CAM technology. Production is based on scan data from a scanned abutment tooth or stone die which is processed with CAD software. The computer-designed substructure is milled either from presintered zirconia blanks (soft milling) followed by densely sintering or from fully sintered blanks (hard milling) in hot isostatic pressed (HIP) zirconia. The surface of the zirconia substructure may then be veneered with silicate-based porcelain [3].

A few studies have been published on the clinical performance of zirconia restorations. For single crowns, Groten and Huttig have presented a 2-year survival rate of 98% [6]. For 5-year follow-ups, Örtorp et al. found a survival rate of 88.8% for 205 crowns [7], Güncü et al. reported 98.1% survival rate for 618 crowns, and Dogan et al. reported a 100% survival rate for 20 anterior placed crowns [8, 9]. Veneered zirconia has also been shown to be a durable and suitable alternative to conventional metal-ceramic, short-span restorations in the anterior and posterior regions [10–14]. However, the most frequent clinical complication found in zirconia FDPs has been chipping of the ceramic veneer [15–17], notably on implant-supported restorations [18, 19]. According to a systematic review, the 5-year survival rate for zirconia FDPs is 90.1% [16]. However, the 5-year survival rates may range from 74.7% to 100% [11, 20]. For longer-term follow-up studies, Ioannidis and Bindl reported a 10-year survival rate of 85% and Håff et al. reported 94% survival after up to 13 years [17, 21]. These studies are often based on a relatively modest study size and are normally carried out in dental practices affiliated with a university, which may give a somewhat skewed conception of an ordinary, typical patient group.

The aim of this three-year retrospective follow-up study was to evaluate the success and survival rates of a large number of ceramic-veneered zirconia FDPs in private practices, manufactured from the same materials with uniform methods.

## 2. Materials and Methods

### 2.1. Study Design

This three-year retrospective follow-up study was essentially compiling and analysing records from all patients consecutively treated with zirconia FDPs. Single crowns and implant-supported FDPs were not included. All FDP treatments were performed between June 2003 and April 2007, by three experienced clinicians at three private practices in Sweden. The criteria for choosing zirconia FDPs were according to the zirconia manufacturer's requirements. Case records from 151 patients (59 men, mean age 58, and 92 women, mean age 58) were analysed. They were treated with a total of 184 zirconia FDPs, and the distribution between the three clinics was 39, 56, and 89 FDPs. The records were evaluated for two months in late 2010 by two of the authors. Factors including age, sex, number of units (abutment teeth and pontics), occluding teeth in the opposing jaw, X-ray status, type of cement used, endodontic treatment before FDP delivery, post material, and prosthetic complications such as cohesive fractures, adhesive fractures, and loss of retention were registered. All other complications during the follow-up period were also registered. The treating dentist conducted clinical follow-up examinations once a year for three years. Patients were asked to contact the clinic if they experienced any problems with their FDP or abutment teeth. The examinations consisted of a complete dental and oral hygiene assessment, including examination of radiographs, bridge stability, and a full cariological and periodontal evaluation.

Of the 458 abutment teeth, 167 were root filled and 134 were treated with posts (48 with indirect technique and 86 with direct technique) before the cementation of FDP.

A zirconia FDP that during the entire observation period remained unchanged, free from complications, and not requiring any intervention, was regarded as* success* [22].

A zirconia FDP that remained in situ over the full observation period and only displaying minor modifications that did or did not require intervention, was regarded as* survival* [22].

A zirconia FDP that was remade due to loss of retention, fracture or extraction of an abutment tooth, fracture of core material, adhesion fracture, extensive cohesion fracture, or pain, was regarded as* failure*.

### 2.2. Prosthodontic Procedures/FDP Manufacturing

**Figure 1:**
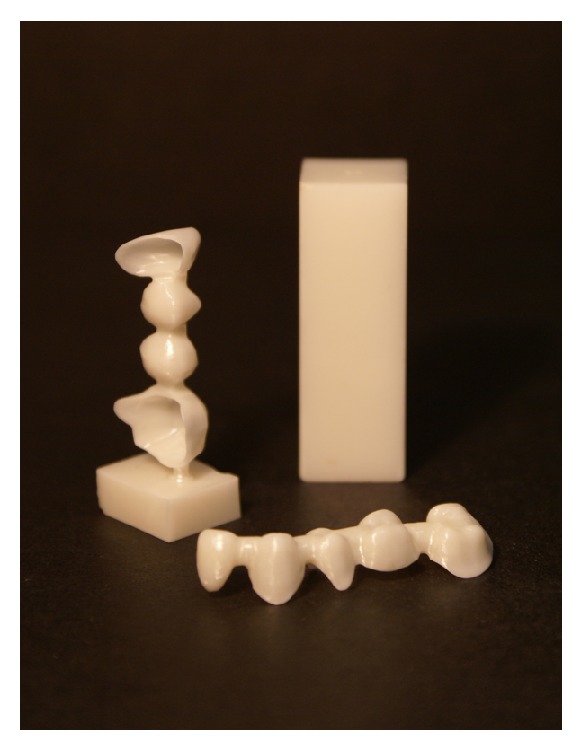
*Right*. Zirconia blank.* Left*. Direct from machining of FDP framework.* Front*. Zirconia FDP framework designed using a CAD program, ready for veneering (Denzir, Cad.esthetics AB, Skellefteå, Sweden).

Abutment teeth were prepared according to the manufacturer's guidelines (Denzir®, Cad.esthetics AB, Skellefteå, Sweden). All FDPs were manufactured by one dental laboratory by two experienced dental technicians according to the manufacturer's instructions. Three dental scanners were used for framework design during the inclusion period (Cad.esthetic system, Cad.esthetics AB, Skellefteå, Sweden; etkon™, Straumann, Basel, Switzerland; 3shape Dental System™, 3shape A/S, Copenhagen, Denmark). The connector dimensions were at least 9 mm^2^, the core thickness was a minimum of 0.5 mm, and the maximum pontic span length was 15 mm. All frameworks were milled at a production center (Denzir, Cad.esthetics AB, Skellefteå, Sweden), from fully sintered, hot isostatic pressed (HIP) zirconia (Figure 1). Before veneering, the frameworks were grinded slightly with diamond burs using a high-speed air turbine handpiece under water irrigation, followed by sandblasting with 50 *µ*m aluminium oxide at 300 kPa pressure, and steam cleaning. The dimensions for the veneering ceramics (GC Initial Zr, GC Europe, Leuven, Belgium) were between 0.2 and 2 mm.

For most of the FDPs (*n* = 109; 59%), a dual-curing, self-adhesive resin luting cement (RelyX™ Unicem, 3M ESPE AG, Seefeld, Germany) was used. Two dual-curing resin luting cement types (Panavia F 2.0, Kuraray America Inc., New York, USA, or Lute it®, Pentron Clinical, Orange, USA) were used for 69 (38%) and three (1.7%) of the FDPs, respectively. A resin modified glass ionomer luting cement (FujiCEM, GC Europe N.V., Leuven, Belgium) was used for one FDP, and for two FDPs the cement type was not registered.

### 2.3. Statistical Analysis

The cumulative success and survival rates (CSR) were calculated according to actuarial life table techniques, and standard errors were calculated using Greenwood's formula.

The data was analysed by means of the Kaplan-Meier survival probability method, and graphs for survival and failure were plotted. Further, a Cox regression analysis was performed on the data for success and survival to investigate the possible influence of different variables. The variables included in the Cox regression model were operator, number of units, being anterior/posterior, maxilla/mandible, cement type, endodontic treatment in abutment tooth, presence of post, and cantilever. Statistical analysis was conducted using SAS version 9.3 (SAS Institute Inc., Cary, USA). The significance level was set at *P* < 0.05. 

## 3. Results

### 3.1. Case Record Evaluation

This study included 151 patients with 184 FDPs. The 184 FDPs consisted of 692 units (458 abutment teeth, 234 pontics). The mean numbers of abutment teeth per FDP and pontics per FDP were 2.5 (range 1 to 6) and 1.3 (range 0 to 4), respectively. Out of 184 FDPs, 21 were cantilever FDPs, with one cantilever pontic each.

Most FDPs (*n* = 143; 78%) were in the posterior region and 117 FDPs (64%) were in the maxilla (Table 1). Eighty-six FDPs were short-span (2-3 units), and 98 FDPs were long-span (4–8 units) (Table 2). All patients had natural teeth or FDPs in the opposing jaw. A total of 37 patients with 45 FDPs were lost to follow-up (Table 3).

### 3.2. Complications

In total, 32 FDPs in 31 patients (17.4% of FDPs, 20.5% of patients) experienced some type of complication (Table 4). Out of these, minor complications such as sensitivity, phonetic problems, untreated pulpitis, and hygiene problems solved by polishing comprised 3.8% of the FDP complications (4.6% of patients).

**Figure 2:**
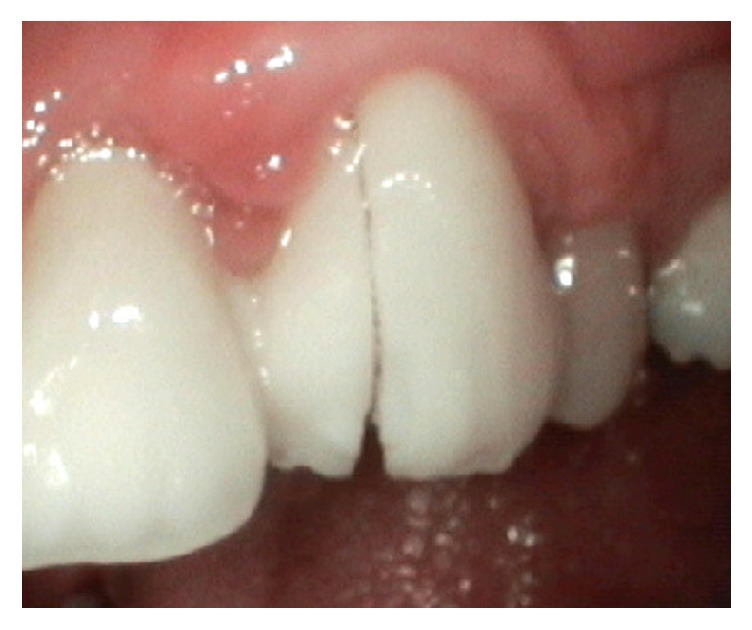
Core fracture found in a six-unit zirconia FDP after one year in situ.

Ceramic fractures occurred in 7.6% of the FDPs. Core fractures occurred in two (1.1%) of the FDPs. One core fracture was seen in a six-unit (five abutments and one pontic) anterior FDP on a bruxist patient (Figure 2). The second core fracture occurred in the median line of a seven-unit (teeth 16–21) FDP.

**Figure 3:**
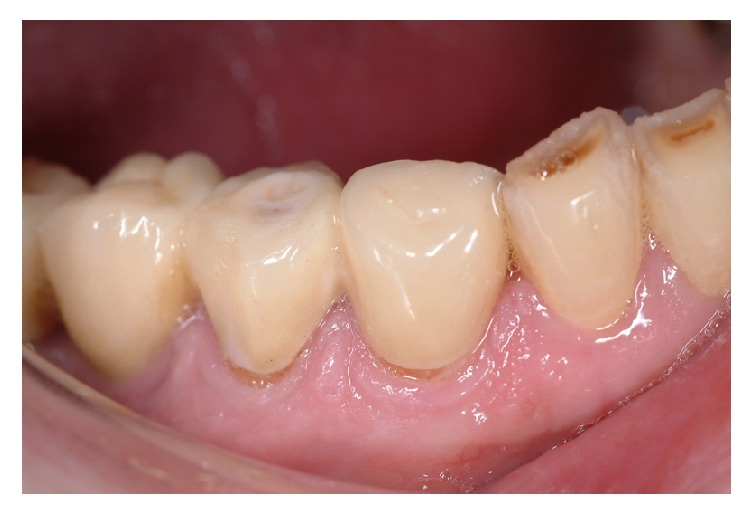
Adhesive fracture during first year due to severe attrition. This FDP was remade.

Adhesive veneer fractures were found in two (1.1%) of the FDPs or 0.6% of all units and ten (5.4%) FDPs had cohesive veneer fractures (1.6% of all units) (Figure 3). Four of these FDPs were remade (in one FDP one unit was replaced for a crown), three FDPs were polished, three repaired with composite or bonded ceramics, and two were left untreated.

According to the definition of success, 25 (13.6%) of 184 FDPs were considered failures. The reasons were as follows: cohesive fractures of the ceramic veneer (10), adhesive fractures (2), core fractures (2), secondary caries (2), extraction of abutment teeth (2), endodontic problems (6), and minor hygiene problems related to the FDP (1). Seven of these FDPs were remade, and two were replaced with implant-supported restorations.

According to the definition of survival, seven (3.8%) FDPs were considered failures. The reasons were as follows: adhesive fracture of ceramic veneer (1), cohesive fracture in combination with insufficient aesthetics and phonetic problems (1), repeated cohesive fractures (1), complications related to endodontic treatments (2), and extraction of abutment teeth (2).

The complications were evenly distributed over time and occurred equally between the three dental practices. The core fractures occurred among long-span FDPs whereas the ceramic veneer fractures where found in both short- and long-span FDPs.

**Figure 4:**
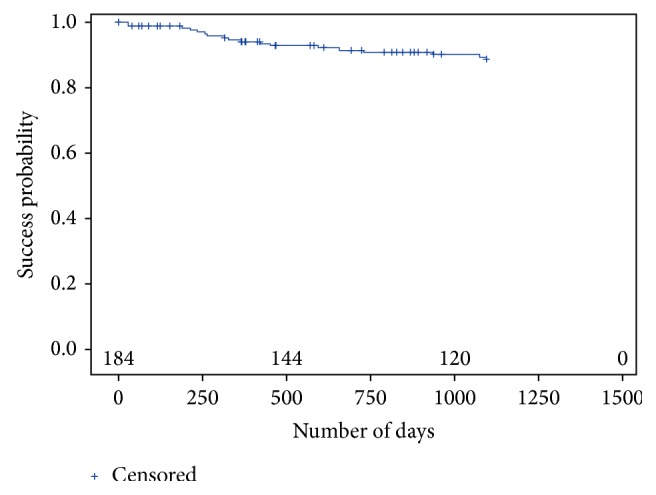
Kaplan-Meier plot according to the definition for success.

**Figure 5:**
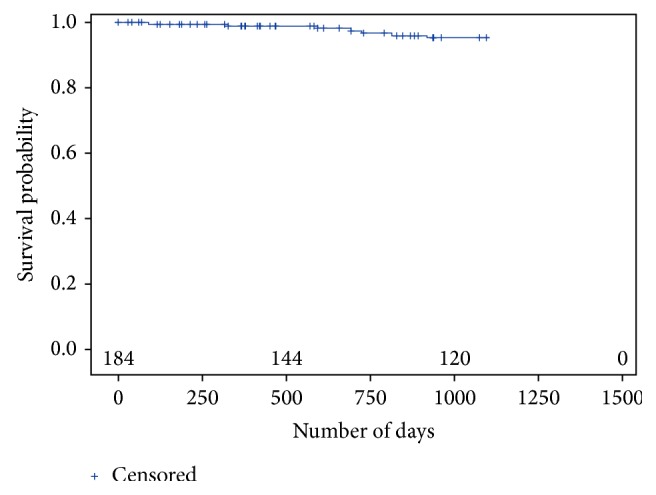
Kaplan-Meier plot according to the definition for survival.

The three-year CSR for success and survival was 82.3% and 95.2%, respectively (Tables 5 and 6). The results from the Kaplan-Meier analysis are presented in Figures 4 and 5.

The output of the multiple Cox regression model for success and the model for survival showed no statistically significant differences for any of the included independent variables, but a tendency towards significance (*P* = 0.0899) was seen for the variable* cement type*. Additionally, a forward stepwise Cox regression was run for both success and survival definitions. A statistical significance was then found for* number of units* (*P* = 0.033) and* cement type* (*P* < 0.0001).

## 4. Discussion

In this study, patient treatment with ceramic-veneered zirconia FDPs, mainly in the posterior region, functioned well during the three-year period, although maintenance was required. Complications occurred in 17.4% of the FDPs. A review of metal-ceramic FDPs at a 1–4-year follow-up reported a 20% mean complication incidence [23]. The single largest problem in the present study was related to ceramic fractures, and ceramic core fractures were seen in two (1.1%) FDPs. Adhesive ceramic veneer fractures were found in two (1.1%) FDPs, and cohesive ceramic veneer fractures occurred in 12 FDPs (6.5%). Core fractures are rare, but according to a systematic review 2.1% of zirconia FDPs suffer core fractures after five years [16]. The reasons for the core fractures in this study were not easily analysed, but one patient was a bruxist, and both core fractures occurred in long-span FDPs. Although the dental laboratory designed the frameworks with a 9 mm^2^ connector dimension, one of the core fractures was seen in the connector area. Several studies on zirconia FDPs have concluded that chipping of the ceramic veneer is a reoccurring problem. Some studies report chipping in 13–32% of zirconia FDPs [10, 14, 24–26], while others report chipping in less than 7% of FDPs in three- to five-year follow-ups [13, 20, 27]. The present three-year study corroborates the latter, lower level of chipping. This confirms the importance of correct dimensioning (maximum 2 mm porcelain) and of following the manufacturer's recommendations in the production process. In this study, the loss of retention was very low (1.1%), probably due to following the manufacturer's instructions for preparation and cementation. Three- to five-year follow-up studies report loss of retention for 3–6% of zirconia FDPs [20, 26, 27].

The cumulative survival rate in this study was 95.2%. In another three-year follow-up, on 17 zirconia FDPs, a 100% survival rate was reported [28]. A four-year follow-up reported a survival rate of 73.9%, and five-year follow-ups have reported 94–100% [26, 29, 30].

The success rate of zirconia FDPs has not been reported as often as the survival, but in this study the three-year success rate was 82.3%; in other studies the success rate may vary from 71% in a three-year follow-up to 89% in a five-year follow-up [28, 30]. Even longer-term follow-up studies have reported success rates, a seven-year follow-up reported 88.8% success, and Håff et al. reported 73% success up to 13 years [21, 31].

It is important to note that HIP zirconia is only one type of zirconia material available on the market. It is not possible to transfer the outcome from this study directly to non-HIP zirconia materials, because of the specific properties of the material. No comparative clinical follow-up studies are currently available for the different types of zirconia and their respective properties.

Patient loss to follow-up (25%) was high with respect to the three-year observation period and was affected since one dentist treated many patients from a neighbouring country and did not attend the recalls. This could also have affected the results by means of an over- or underestimation of the data. The FDPs were fabricated in one laboratory, and the material is uniform; that is, the same zirconia core material and veneering material have been used. The manufacturing processes were carried out by a limited number of operators. The study was based on data from three private practices experienced in ceramic restorations. Being a practice-based study, it has the advantage of being set in a general dental setting rather than in a university clinic [32]. Accordingly, the material examined in this study was extensive in terms of both sample size and spectrum, including both short- to long-span FDPs and FDPs placed in the anterior and posterior regions in both jaws. However, one limitation in this study is the absence of a control group. Larger, long-term retrospective and prospective clinical studies are needed on the performance of zirconia FDPs and the properties and behaviour of the materials over time and on the processing techniques.

The multiple Cox regression showed no statistical significance, which was expected, since there were only a few events (less than 10 events per variable). Interestingly, a tendency towards significance for the type of cement was noted. The additional stepwise Cox regression was consequently used as a supplement to the multiple regressions. The outcome of the stepwise regression showed significance for the type of cement and the number of units. It was on the other hand not possible to see which specific cement influenced the most. However, most of the FDPs (*n* = 109; 59%) were cemented with RelyX and 15.6% of those FDPs experienced complications. Panavia F 2.0 was used for 69 (38%) FDPs and 24.6% had complications. In contrast, Lute it was used for three (1.7%) of the FDPs and two (66.7%) had complications, and FujiCEM was used for one FDP which did not have any complications. The lack of a standardized clinical protocol, which is a drawback with the retrospective study design, makes it difficult to draw any conclusions about the cement. The same should be stated regarding the ceramic chipping; without a rigorous evaluation of the FDPs and a possibility of comparing the anatomy with the baseline FDP, many chippings might pass unreported in the patient charts. An intraoral scanner and a 3D comparison software could be used to match the scans from baseline to follow-up exams, as was done by Selz et al. [33]. Regarding the length of the FDPs, 24.4% of the short-span FDPs reported complications and 16.3% of the long-span FDPs had complications. This result is interesting, since the opposite should have been expected in respect to earlier studies [31, 34]. Since all-ceramic long-span FDPs are not recommended, perhaps the clinicians were stricter when choosing an all-ceramic FDP for a specific patient. Nevertheless, it might be that the type of cement and length of FDP may influence the survival of zirconia FDPs and are therefore of interest for further investigation.

## 5. Conclusions

Within the limitations of this three-year retrospective follow-up study, where only patient charts were analysed, ceramic-veneered zirconia is a promising alternative to metal-ceramic FDPs, even in the posterior area. However, the higher survival rate of metal-ceramic FDPs should be noted and compared to metal-ceramic FDPs, and a higher incidence of complications should be expected. Over the three years, the most common complication was ceramic fractures. Specifically, 6.5% of FDPs had veneer fractures, and 1.1% showed core fractures. Both dentists and patients must be aware of the risks of complications.

## Figures and Tables

**Table 1:** Distribution of 184 zirconia fixed dental prostheses (FDP), by region. Anterior = all units situated mesial of first premolar. Posterior = at least one unit distal of canine.

Region	Maxilla	Mandible	Total FDP
Anterior	33	8	41
Posterior	84	59	143
Total	117	67	184

**Table 2:** Distribution of 184 zirconia fixed dental prostheses (FDPs), by number of units.

Units	FDPs
2	9
3	77
*Total short-span*	*86*

4	65
5	20
6	10
7	1
8	2
*Total long-span*	*98*

*Total FDPs*	*184*

**Table 3:** Reasons for losses to follow-up over 3 years in 151 patients with zirconia fixed dental prostheses.

	Living abroad	Deceased	Moved/new dentist	No contact	Total
Patients (%)	15 (10)	1 (1)	5 (3)	16 (11)	37 (25)

**Table 4:** Complications related to 184 cemented zirconia fixed dental prostheses (FDPs) in 151 patients. Number of incidences (patients within brackets).

Complications	Incidence
Retention loss	2 (1)
Extraction (fracture or endodontic problem)	3 (3)
Root separation	2 (2)
Endodontic problem	8 (8)
Caries (secondary)	2 (2)
Others (phonetic, hygiene, sensitivity)	7 (6)
*Ceramic fractures:*	
Core	2 (2)
Adhesive	2 (2)
Cohesive	12 (9)

**Table 5:** Life table analysis of zirconia fixed dental prostheses (FDPs). Cumulative Success Rate (CSR).

Period (years)	Examined FDPs	Lost to follow-up	Failure^*∗*^	CSR (%)	Standard error
FDP cementation	184	0	0	100	
1 year	156	16	12	91.9	2.2
2 year	135	13	8	86.2	2.9
3 year	118	12	5	82.3	3.2
Total	118	41	25	82.3	

^**∗**^A zirconia FDP that during the entire observation period remained unchanged, free from complications, and not requiring any intervention was regarded as a *success.*

**Table 6:** Life table analysis of zirconia fixed dental prostheses (FDPs). Cumulative Survival Rate (CSR).

Period (years)	Examined FDPs	Lost to follow-up	Failure^*∗*^	CSR (%)	Standard error
FDP cementation	184	0	0	100	
1 year	165	16	3	98.1	1.1
2 year	150	13	2	96.7	1.4
3 year	132	16	2	95.2	1.8
Total	132	45	7	95.2	

^**∗**^A zirconia FDP that remained in situ over the full observation period and only displaying minor modifications that did or did not require intervention was regarded as *survival.*

## Additional Points

*Clinical Significance*. According to this three-year study, the clinical outcome for ceramic-veneered zirconia FDPs was acceptable, even when placed in the posterior area. However, a higher incidence of complications compared to metal-ceramic FDPs should be expected. And 3.8% were judged as failures during the follow-up period.
